# Successful salvage treatment of myxoid liposarcoma with multiple peritoneal seeding using helical tomotherapy-based intraperitoneal radiotherapy: a case report

**DOI:** 10.1186/s13104-015-1134-8

**Published:** 2015-05-02

**Authors:** Chihwan Choi, Ji Hye Park, Chang Geol Lee, Hyun Ju Kim, Chang-Ok Suh, Jaeho Cho

**Affiliations:** Department of Radiation Oncology, Yonsei University College of Medicine, 50 Yonsei-ro, Seodaemun-gu, Seoul 120-752 South Korea; Department of Pathology, Yonsei University College of Medicine, 50 Yonsei-ro, Seodaemun-gu, Seoul 120-752 South Korea

**Keywords:** Myxoid liposarcoma, Whole abdominal radiotherapy, Intensity-modulated radiotherapy, Helical tomotherapy, Radiosensitivity

## Abstract

**Background:**

Myxoid liposarcoma is the most common soft-tissue sarcoma that metastasizes to the peritoneal cavity. Recently, an advanced intensity-modulated radiotherapy, known as helical tomotherapy, has been introduced to improve target coverage, while reducing normal tissue radiation. Here, we report a case of myxoid liposarcoma with multiple peritoneal seeding that was chemotherapy-refractory, but was successfully salvaged by helical tomotherapy-based intraperitoneal radiotherapy.

**Case presentation:**

A 71-year-old East-Asian male was initially diagnosed with myxoid liposarcoma in his left thigh by excision. Six years later, the patient underwent a left pneumonectomy for metastatic myxoid liposarcoma in the left lung. Since then, the patient was treated with two segmental resections, and multiple lines of chemotherapy, for repeated recurrences in the peritoneal cavity. The patient underwent intraperitoneal radiotherapy followed by tumor boost radiotherapy, as salvage treatment for chemotherapy-resistant metastatic peritoneal myxoid liposarcoma. The prescribed dose was 24 Gy delivered in 15 fractions of 1.6 Gy over 3 weeks, followed by a 16 Gy boost dose administered in eight fractions of 2 Gy, to multifocal peritoneal lesions. A positron emission tomography scan obtained 8 weeks after completion of radiotherapy, showed a complete metabolic response of metastatic peritoneal lesions. Radiotherapy was well tolerated, without any side effects. In a computed tomography scan obtained 20 weeks after completion of radiotherapy, most of the peritoneal metastatic lesions had disappeared, except for two small residual nodules.

**Conclusion:**

This case suggests that low fraction-sized intraperitoneal radiotherapy (1.6 Gy administered once daily), followed by a focal boost using helical tomotherapy, is a feasible treatment without side effects. It produced an excellent tumor response, and durable intraperitoneal control for metastatic peritoneal myxoid liposarcoma.

## Background

Liposarcomas are malignant tumors of mesenchymal origin that arise from adipose tissue, and are the second most frequent type (comprising 15–20%) of all soft tissue sarcomas (STS), with incidence peaks in the fourth to sixth decades of life [1-3]. The World Health Organization recognizes four subtypes: well-differentiated, dedifferentiated, myxoid/round cell, and pleomorphic. Myxoid liposarcomas (MLS) tend to be low grade, and to metastasize less frequently than the other types of liposarcoma. Unlike other types of liposarcoma, however, MLS has a high potential for extrapulmonary metastasis [4-6]. Peritoneal metastases are considered an uncommon event in the natural history of STS. They may denote refractory disease, and can cause bowel obstruction, bleeding, and perforation. Limited information is available regarding the treatment of peritoneal metastasis from non-peritoneal primary STS. MLS is the most common sarcoma that metastasizes to the peritoneal area [7]. For patients with progressive metastatic peritoneal disease, after undergoing treatment with multiple lines of chemotherapy and several surgical interventions, the therapeutic options are very limited, and are often restricted to best supportive care only.

The excellent tumor control from whole abdominal radiotherapy (WART) has been reported in ovarian cancer patients with peritoneal seeding or carcinomatosis [8-11]. However, conventional WART is a non-optimal technique because it does not sufficiently cover the peritoneal cavity while sparing at-risk organs, such as the kidneys, liver, and bone marrow. Recently, advanced intensity-modulated radiotherapy (IMRT) techniques, such as helical tomotherapy (HT) and volumetric modulated arc therapy (VMAT), have been introduced to improve target coverage, while reducing normal tissue damage. If WART using IMRT techniques could spare the organs at risk, such as the kidneys, liver, and bone marrow, it would be a misnomer. Therefore, we have defined WART using IMRT techniques as intraperitoneal radiotherapy (IPRT), because HT and VMAT focus more on intraperitoneally seeded tumors, while avoiding major dose-limiting organs in the abdomen. Here, we report a case in which low fraction-sized IPRT using HT produced an excellent tumor response and control for MLS with multiple peritoneal seeding, without treatment-related side effects.

## Case presentation

**Figure 1:**
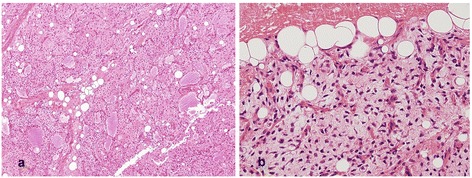
The patient was initially diagnosed with a myxoid liposarcoma in his left thigh by excision in 2000. A photomicrograph of the tumor shows small proliferating spindle cells and lipoblasts in the myxoid stroma, findings consistent with myxoid liposarcoma. **(a)** Hematoxylin and eosin stain (40X magnification). **(b)** Hematoxylin and eosin stain (200X magnification).

**Figure 2:**
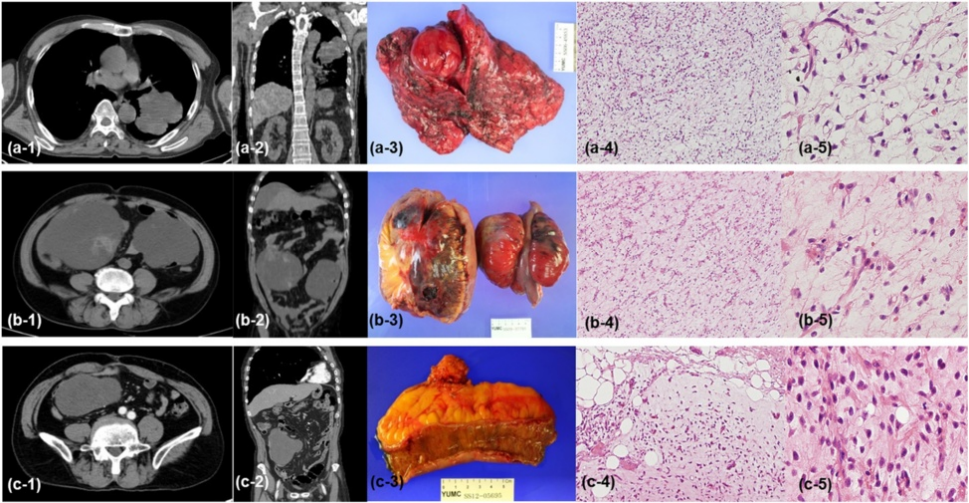
Transverse **(a**-**1**, **b**-**1**, and **c**-**1**) and coronal **(a**-**2**, **b**-**2**, and **c**-**2** **)** computed tomography, macroscopic **(a**-**3**, **b**-**3**, and **c**-**3** **)**, and microscopic images (40X: d, i, and n, 200X: e, j, and o) of metastatic myxoid liposarcoma. **(a**-**1**–**a**-**5)** Images from December 2006, when the patient underwent a left pneumonectomy for myxoid liposarcoma metastasizing to the left lung.** (b**-**1**–**b**-**5)** Images from August 2009, when the patient underwent segmental resection for a peritoneal metastatic myxoid liposarcoma, involving the mesentery and subserosa of the jejunum. **(c**-**1**–**c**-**5)** Ever since then, the patient was treated by one additional segmental resection for a recurred peritoneal metastatic myxoid liposarcoma in February 2012.

**Figure 3:**
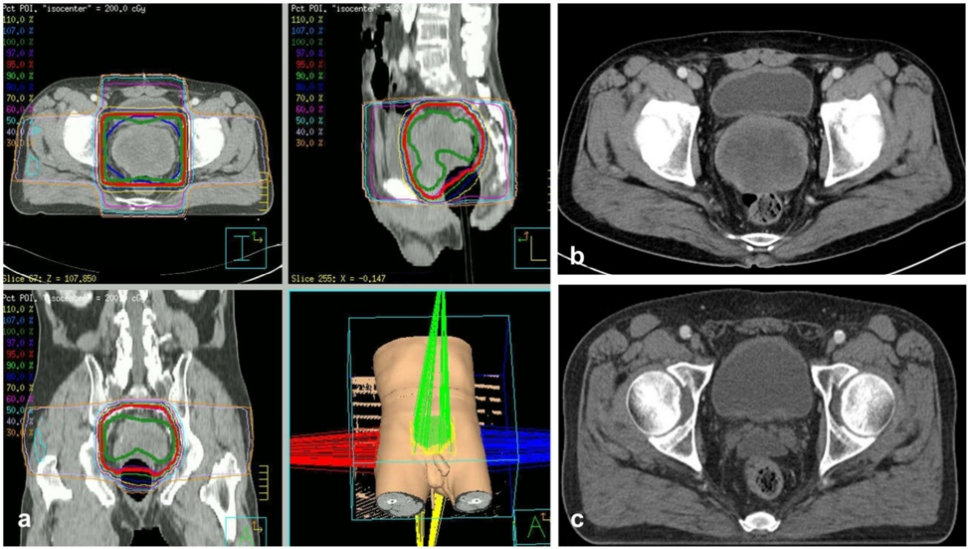
The three-dimensional radiotherapy for the rectal-shelf mass **(a)** Dose distribution for the three-dimensional radiotherapy plan for the metastatic myxoid liposarcoma, with a maximum size of 9.1 cm in the rectovesical pouch. The patient underwent salvage radiotherapy at a dose of 40 Gy delivered in 20 fractions of 2 Gy over 4 weeks between September and October 2012. **(b)** A computed tomography scan, obtained in November 2012 after completion of radiotherapy, showed that the metastatic mass had decreased by 6.9 cm in size. **(c)** A computed tomography scan, obtained in May 2013, showed that the mass had decreased by 4.0 cm in size.

**Figure 4:**
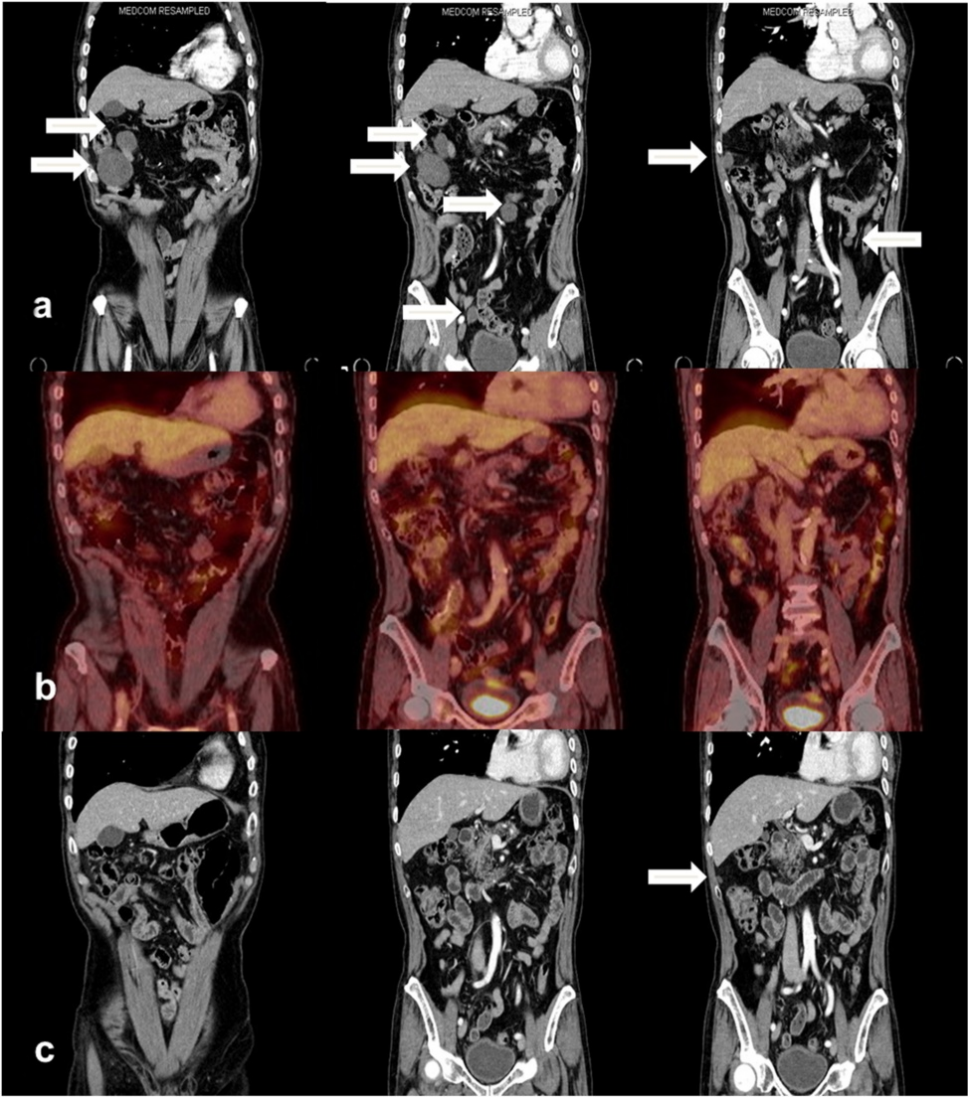
Coronal images of computed tomography or positron emission tomography scans showing a regression of peritoneal metastases. **(a)** Contrast-enhanced abdominal computed tomography scan showing multifocal metastatic peritoneal nodules (white arrows) in September 2013. The patient completed intraperitoneal radiotherapy followed immediately by tumor boost radiotherapy. **(b)** Surveillance by positron emission tomography scan, obtained 8 weeks after completion of radiotherapy, showed a complete metabolic response of metastatic peritoneal lesions. **(c)** A contrast-enhanced computed tomography scan, obtained in March 2014 after completion of radiotherapy, shows that most of the metastatic peritoneal lesions had disappeared.

**Figure 5:**
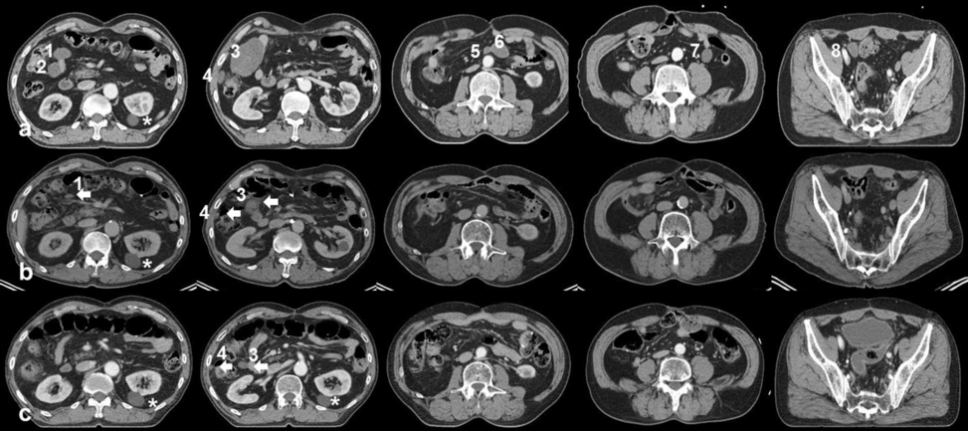
Transverse images of a contrast-enhanced abdominal computed tomography scan showing a regression of the peritoneal metastases. **(a)** An abdominal computed tomography scan from September 2013, showed multifocal metastatic peritoneal nodules (numbered from ‘1’ to ‘8’) before intraperitoneal radiotherapy. **(b)** Surveillance by computed tomography scan, obtained in December 2013 after completion of radiotherapy, showed a complete response of five nodules (1, 2, 5, 6, 7, and 8) and a partial response of three nodules (1, 3, and 4). **(c)** A computed tomography scan, obtained in March 2014 after completion of radiotherapy, showed that most of the metastatic peritoneal lesions had disappeared, except two small nodules that had decreased in size by about 0.8–1 cm (3 and 4). The asterisk (*) indicates a simple benign cyst.

In September 2013, a 71-year-old East-Asian male visited our institution with a 5-month history of abdominal pain, nausea, and constipation. Physical examination revealed a distended abdomen due to multiple, non-tender masses, with no signs of peritonitis or abdominal wall hernias. The Eastern Cooperative Oncology Group (ECOG) performance status of the patient was “1”. His medical history included a huge, non-tender, deep, rubbery MLS (pT2bN0M0, positive resection margin), with a maximum size of 20 cm at the medial side of the left thigh. The tumor had been treated by excision (Figure 1) and postoperative RT at a dose of 63 grays (Gy) delivered in 35 fractions of 1.8 Gy over 7 weeks in 2000. Six year later in 2006, the patient underwent a left pneumonectomy for a MLS metastasizing to the left lung (Figure 2 a-1–a-5). The removal was radical, with free margins and an intact tumor capsule. Postoperatively, no adjuvant therapy was indicated, and close follow-up was recommended. In August 2009, the patient underwent segmental resection for two peritoneal metastatic nodules (size: 15 × 15 × 11 cm and 14 × 9 × 9 cm, respectively) involving the mesentery and subserosa of the jejunum (Figure 2 b-1–b-5), and adjuvantly received 6 cycles of chemotherapy with adriamycin and ifosfamide. Since then, one additional segmental resection was performed for a recurrent peritoneal metastatic MLS (size: 13 × 13 × 7 cm) in February 2012 (Figure 2 c-1–c-5). Abdominal computed tomography (CT) imaging in May 2012 showed a progressive mass in the rectovesical pouch. It caused symptoms of abdominal pain and constipation. The patient received two cycles of chemotherapy with gemcitabine and docetaxel, followed by two cycles of dacarbazine. Despite this chemotherapy, the rectovesical pouch mass progressed. The patient refused any further chemotherapy. He was referred for salvage RT of his progressive, rectal-shelf mass. The radiation prescribed for the rectal-shelf mass was 40 Gy, to be delivered in 20 fractions of 2 Gy over 4 weeks, between September 2012 and October 2012 (Figure 3). No acute skin or gastrointestinal toxicity was reported. This treatment provided an excellent tumor response, and symptom relief for the patient. The tumor mass slowly regressed over the course of a year.

**Figure 6:**
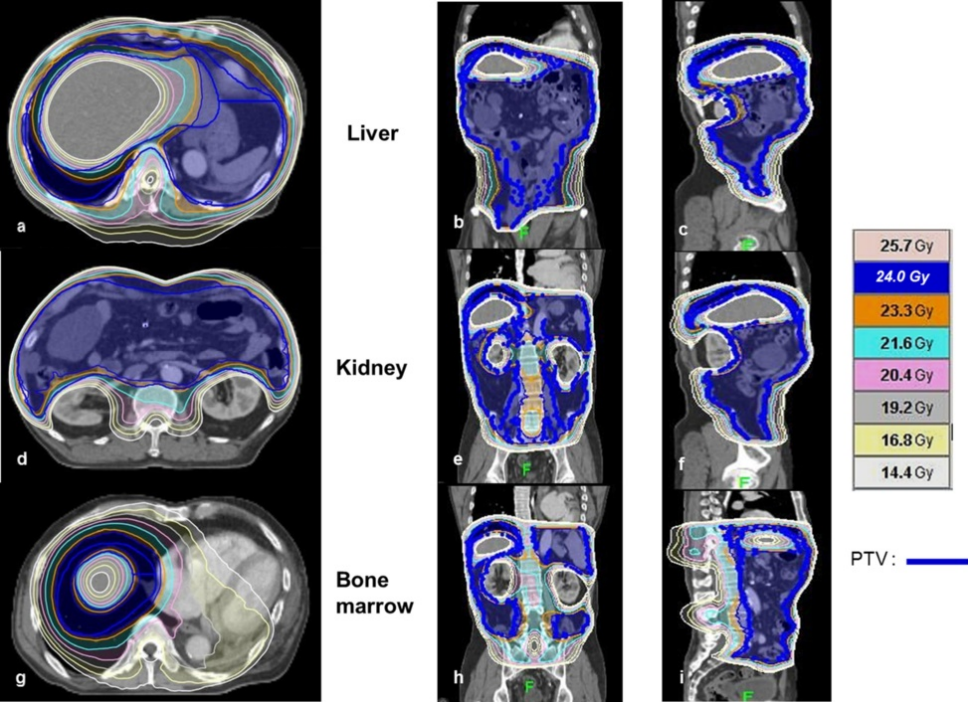
Radiation dose distribution for intraperitoneal radiotherapy using helical tomotherapy-based intensity-modulated radiotherapy, with particular attention to representative dosimetry within the liver **(a**-**c)** bilateral kidneys** (d**-**f)**, and bone marrow **(g**-**i)**. Transverse **(a**, **d**, and **g)**, coronal **(b**, **e**, and **h)**, and sagittal computed tomography images **(c**, **f**, and **i)** with isodose radiation color contour maps are provided.

**Figure 7:**
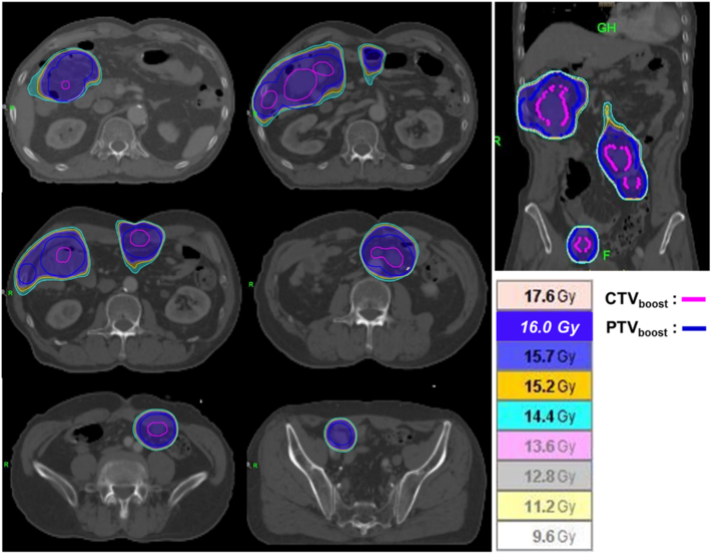
A boost radiotherapy dose distribution in the coronal plane. The gross tumor volume was equal to the clinical target volume. The planning target volume was made by a three dimensional expansion of the clinical target volume_,_ with 0.5 cm axial and 1 cm superior/inferior margins.

However, follow-up abdominal CT in September 2013, showed multifocal peritoneal nodules with fatty components within the mesentery of the jejunum, ileum, and ascending colon (Figures 4 a and 5 a). The patient refused further salvage chemotherapy, but gave informed consent for treatment with IPRT using HT. A dose of 24 Gy was prescribed, to be delivered in 15 fractions of 1.6 Gy over 3 weeks, followed by a 16 Gy boost dose in 8 fractions of 2 Gy delivered on each residual metastatic peritoneal lesion. The clinical target volume (CTV) was defined as the whole peritoneal cavity, supplemented by the aortocaval and iliac nodes, as described by Duthoy *et al*. [10]. The following at-risk organs were delineated: the liver, both kidneys, and the spine. An IPRT dose distribution in the transverse and coronal plane is illustrated in Figure 6. Average doses to the liver, and right and left kidneys were 16.9 Gy, 13.8 Gy, and 14.1 Gy, respectively, which are sufficiently lower than the limiting doses. The second CT scan was used to generate an adaptive HT-based IMRT plan, which recommended a 16 Gy boost in 8 fractions of 2 Gy for residual multifocal peritoneal lesions that had shrunken after IPRT. The residual tumor was defined as the gross tumor volume (GTV_boost_). For second treatment planning, the GTV_boost_ was equal to the CTV_boost._ The planning target volume (PTV_boost_) was made by three-dimensional expansion of the CTV_boost_ with 0.5 cm axial, and 1 cm superior/inferior margins. A RT boost dose distribution in the coronal plane is illustrated in Figure 7. The patient completed IPRT, followed by tumor boost RT, between September 2013 and October 2013.

Clinical symptoms and treatment-related side effects were monitored weekly during treatment, at the end of treatment, and monthly thereafter. RT was well tolerated, without any side effects, such as bone marrow suppression, nausea, diarrhea, or abdominal cramps. The patient did not experience tumor lysis syndrome. Surveillance by positron emission tomography (PET) scan in December 2013 showed a complete metabolic response of the peritoneal metastatic lesions (Figure 4 b). Furthermore, the CT scan obtained the same day showed a complete response for 5 nodules and a partial response for the 3 others (Figure 5 b). The ECOG performance status of the patient remained “0”. The patient, who had initially presented with abdominal pain, nausea, and constipation, obtained the return of peristalsis, normal food intake, and normalization of stools. In a CT scan obtained in March 2014, 5 months post completion of RT, most of the peritoneal metastatic lesions had disappeared, except for two small nodules that had decreased to 1.0 cm and 0.8 cm from 7.2 cm and 2.4 cm in size, respectively (Figures 4 c and 5 c). The patient has not developed late hematologic, genitourinary, or gastrointestinal toxicity within the 9 months post RT.

## Discussion

Using the case presented here, we report on the effective salvage of recurrent chemotherapy-resistant peritoneal lesions after segmental resection for a metastatic peritoneal MLS, by IPRT using HT. Although some studies have suggested that MLS tends to be relatively more radiosensitive than other types of STS, with high rates of regression and even complete clinical response [12,13], RT, covering the whole intraperitoneum, has not been well established as a salvage treatment in metastatic peritoneal MLS. However, it has been reported that conventional WART doses between 20 and 30 Gy produce high tumor response rates of 50–70% in the treatment of epithelial ovarian carcinoma [14,15]. With this in mind, we exploited IPRT using HT for this challenging case of metastatic chemotherapy-resistant peritoneal MLS.

Limited data are available on the treatment for metastatic peritoneal relapse from MLS. Surgery may be the treatment of choice for those patients with complications, such as incomplete intestinal obstruction, abdominal pain, abdominal distension, gastrointestinal bleeding, urinary obstruction, and anorexia [16,17]. However, given the reports of repetitive peritoneal relapse following surgical excision, treatment with re-excisions may not be appropriate. For high-grade or recurrent or metastatic lesions, conventional chemotherapy with ifosfamide and doxorubicin may be indicated [18,19,17,16]. In a recent randomized controlled phase III trial [18], the median progression-free survival was significantly higher for the combination arm using doxorubicin and ifosfamide than for the doxorubicin alone arm (7.4 vs 4.6 months; p = 0.003). The combination arm also had a higher overall response rate than the doxorubicin alone arm (26% vs. 14%; p < 0.0006). Recently, a marine-derived compound, trabectedin, has been introduced, and is believed to be effective [17,16,20]. In a prospective phase II study of neoadjuvant trabectedin in patients with advanced localized myxoid liposarcoma [20], the objective response rate was 24% and no disease progression was reported. The use of intraoperative radiotherapy (IORT) has provided encouraging results in patients with primary and recurrent retroperitoneal STS [21-23]. A protocol involving maximal tumor resection and IORT, with or without external beam RT, might be effective for local control in patients with metastatic peritoneal relapse from MLS.

Conventional WART has been effective in intraperitoneal tumor control to some degree. However, it would not have sufficiently covered the whole intraperitoneal cavity, as we wished to spare the liver and kidneys using the basic technique of anterior to posterior (AP) and posterior to anterior (PA) direction beam delivery. Modern advances in RT techniques, such as IMRT, have allowed us to overcome the shortcomings of conventional WART [10,24]. A retrospective study showed that IPRT, delivered using IMRT, could offer significant palliation in the case of peritoneal metastatic ovarian cancer [11]. IPRT using HT provides superior target coverage, while simultaneously dose-limiting critical at-risk organs, when compared to conventional WART. HT can improve radiation dose coverage of peritoneal surfaces, especially near the kidney and liver, due to the freedom of beam direction and delicate intensity modulation. HT can also deliver lower radiation doses to the liver, bilateral kidneys, and bone marrow [25]. The broad practical use of IPRT is limited by an extended radiation beam field lengths of >40 cm, multiple dose isocenters in the abdomen and pelvis, and treatment complexity. Compared to other IMRT techniques, IPRT using HT can obviate extended beam field lengths and multiple dose isocenters owing its continuous helical delivery of radiation. Taking all these facts into consideration, we believed that IPRT using HT could offer effective symptom relief and tumor control in the case of peritoneal metastatic MLS, without significant hematologic, genitourinary, or gastrointestinal toxicity.

No consensus currently exists regarding the optimum IPRT dose and fractionation schedule for metastatic peritoneal lesions from MLS. Future research should focus on obtaining the lowest radiation dose that induces symptomatic relief without significant toxicity. Perhaps a more modest dose might obtain the same palliation as reported in the present study. In our case, the low fraction-sized IPRT schedule (1.6 Gy given once daily) appears to be associated with the lack of any side effects experienced by the patient, such as bone marrow suppression, nausea, diarrhea, or abdominal cramps. Although there is an understanding of the mechanism of cell death by radiation at conventional fraction size (1.8–3.0 Gy per fraction), the effect and safety of IPRT at a lower fraction size (<1.8 Gy) on patients with a peritoneal metastatic MLS is still unknown, and should be further investigated with clinical observation and preclinical studies.

Generally, patients with MLS have a good prognosis, and they have the potential for gaining maximum benefit from techniques that minimize the side effects of RT [13]. MLS primarily metastasizes to extrapulmonary sites, most commonly the peritoneal cavity, in contrast with other STS [4-6]. The low pulmonary metastatic potential of MLS provides the patients with a relatively long survival time [26]. MLS has been reported to be more radiosensitive compared to other STS [12,13]. Therefore, IPRT using HT could be a effective treatment for patients with peritoneal metastatic MLS, although it is most likely to be a palliative procedure. When the therapeutic options are very limited, IPRT using an advanced technique like IMRT, could be a good option for patients with progressive metastatic peritoneal disease after undergoing treatment with multiple lines of palliative chemotherapy, and several surgical interventions.

## Conclusion

The present case report shows that low fraction-sized IPRT (1.6 Gy given once daily), followed by tumor boost RT using HT, provided excellent tumor control and symptom relief for a patient with bulky multifocal abdominal tumors, identified as a metastatic peritoneal MLS. Our findings, taken together with reports from the literature, emphasize that radiosensitivity in other types of cancer could translate into excellent tumor control when IPRT using HT is appropriately applied.

### Ethics statement

This study was performed in accordance with the Helsinki Declaration and the written consent by the patient.

## Consent

Written informed consent was obtained from the patient for publication of this Case Report and any accompanying images. A copy of the written consent is available for review by the Editor-in-Chief of this journal.

## Abbreviations

CT: Computed tomography
CTV: Clinical target volume
ECOG: Eastern cooperative oncology group
GTV: Gross tumor volume
Gy: Gray
HT: Helical tomotherapy
IMRT: Intensity-modulated radiotherapy
IORT: Intraoperative radiotherapy
IPRT: Intraperitoneal radiotherapy
MLS: Myxoid liposarcoma
PET: Positron emission tomography
PTV: Planning target volume
RT: Radiotherapy
STS: Soft-tissue sarcoma
VMAT: Volumetric modulated arc therapy
WART: Whole abdominal radiotherapy
